# Preparation of Gold Nanoplates Using Ortho Carbonyl Compounds as Capping Agents for Electrochemical Sensing of Lead Ions

**DOI:** 10.1186/s11671-021-03521-2

**Published:** 2021-04-07

**Authors:** Xinde Jiang, Jianye Ma, Guixian Jiang, Manqing Xu, Xueping Huang, Guiqing Gao, Xin Dai

**Affiliations:** 1 College of Civil and Architectural Engineering, Nanchang Institute of Technology, Nanchang, 330099 China; 2 High-Tech Development Zone, Tianxiang Road 289, Nanchang, 330099 Jiangxi China

**Keywords:** Nanoparticle, Organic compounds, Electrochemistry, Wastewater

## Abstract

In this study, gold nanoplates were synthesized using plant molecules (gallic acid) following a kinetic control mode. The growth of nanoplates is mainly due to the specific adsorption of capping agents on certain crystal facets. Through systematical characterizations, it is found that the distance between two oxygen atoms in ortho carbonyl compounds matches well with the lattice spacing of gold (111) facets exactly, which is beneficial to the formation of twin seeds and further the growth of plate-like gold nanoparticles. The gold nanoplates on glassy carbon electrode show a remarkably improved electrochemical sensing activity of lead ions compared to the bare glassy carbon electrode or spherical gold nanoparticle-modified electrode. The modified electrode is expected to be used in the detection of lead ion concentration in heavy metal wastewater.

## Introduction

Thanks to the localized surface plasmon resonance (LSPR) property [1–4], gold nanoparticles (GNPs) have found many optical and electrochemical applications, including sensing, Raman spectroscopy, biological imaging, catalysis, biomedicine, and so forth [5–10]. The plasma properties of GNPs depend on their shape, size, composition and dielectric environment; especially, the near-field enhancement of anisotropic GNPs is often highly amplified due to their sharp structural characteristics [11, 12]. In various morphologies, two-dimensional gold nanoplates have attracted much attention due to their unique optical properties, high conductivity, thermal stability and catalytic activity [13–15]. In the past few decades, a series of preparation methods have been developed to synthesize two-dimensional gold nanoplates, including photochemical reaction method, thermal decomposition method, seed-mediated method, microwave-assisted method and ultrasonic-assisted method [16–20]. However, most of these synthesis methods are not environmental friendly as they often involve the use of many surfactants or capping agents (cetyltrimethylammonium bromide, sodium dodecyl sulfate), chemical reducing agents (NaBH_4_), etc. [21–23].

In recent years, the vigorous development of green chemistry has promoted the preparation of gold nanoplates by biological method [24]. Biomass such as lemongrass, *aloe vera*, seaweed, alfalfa, *E. coli* and *Platycladus orientalis* extract has been used as reductive and protective agents to synthesize gold nanoplates [25, 26]. For example, Shankar et al. [27] developed a biological method to produce up to 45% gold nanoplates by citronella leaf extract. Montes et al. [28] successfully prepared anisotropic gold nanoplates with size of 500–4000 nm and thickness of 15–30 nm by reducing HAuCl_4_ solution with the aqueous extract of alfalfa. Zhan et al. [29] reported a new method for the synthesis of gold nanoplates, i.e., the biological reduction of HAuCl_4_ by using *Platycladus orientalis* extract with a kinetic control instrument. It is worth mentioning that the yield of gold nanoplates can be tuned by adjusting the experimental parameters, such as the feeding way/rate of the reagent, or the temperature and pH of the feed solution. For example, when the pH was 2.81 and the temperature was 60 °C, the yield of gold nanoplates could be up to 39% by injecting *Platycladus orientalis* extract into the gold precursor at a speed of 60 mL·h^−1^.

It is difficult to give the exact mechanism of the nucleation and growth of GNPs in biosynthesis because the real active molecules in plant extracts are difficult to distinguish [30]. In previous studies, it was found that polyphenols play an important role in the formation of gold nanoplates [31]. In this study, gallic acid as a representative of polyphenols was used to study the growth mechanism of GNPs. Through a wide spectrum of structural characterizations, the role of ortho carbonyl compounds in the growth of gold nanoclusters into twin seeds and then plate-like nanoparticles was identified, and these as-prepared gold nanoplates were further used in the electrochemical detection of lead ions.

## Material and Method

### Material

Chloroauric acid, gallic acid, sodium oxalate, ascorbic acid, potassium ferricyanide, cadmium dichloride and lead sulfate are all analytical pure and purchased from Aladdin Chemical Reagent Co., Ltd.

### Preparation of Gold Nanoplates

In a typical synthesis of gold nanoplates, a two-neck flask (50 mL) containing 10 mL chloroauric acid (1.0 mM) was preheated in an oil bath (equipped with magnetic stirring) at 30 °C for 5 min. Feed solutions (gallic acid, 0.6 mM, 10 mL) were simultaneously injected into the flask through syringe pump (Shenzhen medical equipment technology development Co., Ltd., SK-500, China) at the addition rate of 0.5, 1.0, 1.5, 2.0 and 2.5 mL·min^−1^, respectively. The reaction mixture was maintained stirring for an additional 30 min after the completion of feeding.

### Characterization

The UV–Vis spectrum of GNPs was measured by UV–Vis spectrophotometer (TU-1900, Beijing Purkinje General Instrument Co., Ltd., China) with water as reference, the scanning wavelength range was 330–1100 nm, and the scanning step length was 1.0 nm. Transmission electron microscopy (TEM), high-resolution transmission electron microscopy (HRTEM), selected area electron diffraction (SAED) and energy-dispersive spectroscopy (EDS) were performed on a Phillips Analytical FEI Tecnai 30 electron microscope (300 kV). Fourier transform infrared spectrometer (FTIR) analysis was carried out by infrared spectrometer (Nicolet iS50, Nicolet company, USA), and the scanning wavenumber range was 400–4000 cm^−1^. Thermogravimetry (TG) analysis was carried out in the thermogravimetry analyzer (TG209F1, Netzsch, Germany). The temperature range was 30–800 °C, the heating rate was 10 °C ·min^−1^, and the air flow rate was 20 mL·min^−1^. XRD measurements for the gold nanoplates were conducted on an X-ray diffractometer (Bruker D8 Advance, Germany) equipped with Cu Ka radiation (40 kV, 30 mA). XPS analysis was performed on a Quantum 2000 spectrometer using the Al-Ka line as the excitation source. Chromatographic separation was performed by means of an Agilent 1290 LC system equipped with a waters cortecs C18 column and a mobile phase consisting of formic acid solution (mixed with water, 10%) and methanol at a flow rate of 0.2 mL/min. The volume of extract injected was 20 μL. The methanol gradient used was as follows: 10% at time (*t*) = 0 min, 10% at *t* = 1 min, 90% at *t* = 8 min, 100% at *t* = 12 min and maintained until *t* = 13 min. MS detection was carried out using a Agilent 6550 mass spectrometer equipped with a heated electrospray ionization source, and all compounds were determined in the negative mode. The yield of the gold nanoplates was calculated by dividing the number of gold nanoplates by the total number of GNPs. In order to ensure the accuracy of the data, the number of nanoparticles analyzed was more than 1000.

### Electrochemical Sensing of Lead Ions

The glassy carbon electrode (GCE, 3 mm in diameter) was polished with 0.3 and 0.05 μm alumina and then washed by ultrasonication for 15 min in ethanol and ultra-pure water, respectively. The as-prepared GNPs sol (100 uL) was drop-casted on the glassy carbon electrode and dried in air. The casting of GNPs was repeated three times. Linear sweep voltammetry test was conducted with glassy carbon electrode (modified with GNPs) as the working electrode, a platinum wire as counter electrode and an Ag–AgCl electrode as reference electrode. The conditions of voltammetric test were: minimum voltage − 2.0 V, maximum voltage 2.0 V and scan rate 1 mV·S^−1^.The concentration of lead ions in the voltammetric test is range from 1000 to 1 mg·L^−1^, and this concentration of lead ions usually appears in pollutant water samples [32, 33].

## Results and Discussion

### Effect of Feeding Rate

**Fig. 1:**
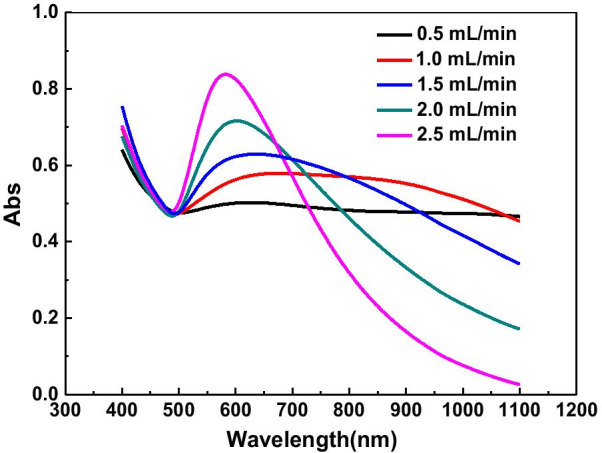
UV–Vis spectra of GNPs prepared with the feeding rate of 0.5, 1.0, 1.5, 2.0 and 2.5 mL·min^−1^

In order to avoid the violent nucleation and growth of GNPs, the feeding rate of gallic acid was controlled by an injection pump, which consequently regulates the release rate of gold atoms during the reduction process. The effect of feeding rate on the yield of gold nanoplates was investigated. As shown in Fig. 1, as the feeding rate decreases, the surface plasmon resonance peak of spherical GNPs gradually decreases, while a new absorption peak appears in the long wavelength region(such as red line in Fig. 1).

**Fig. 2:**
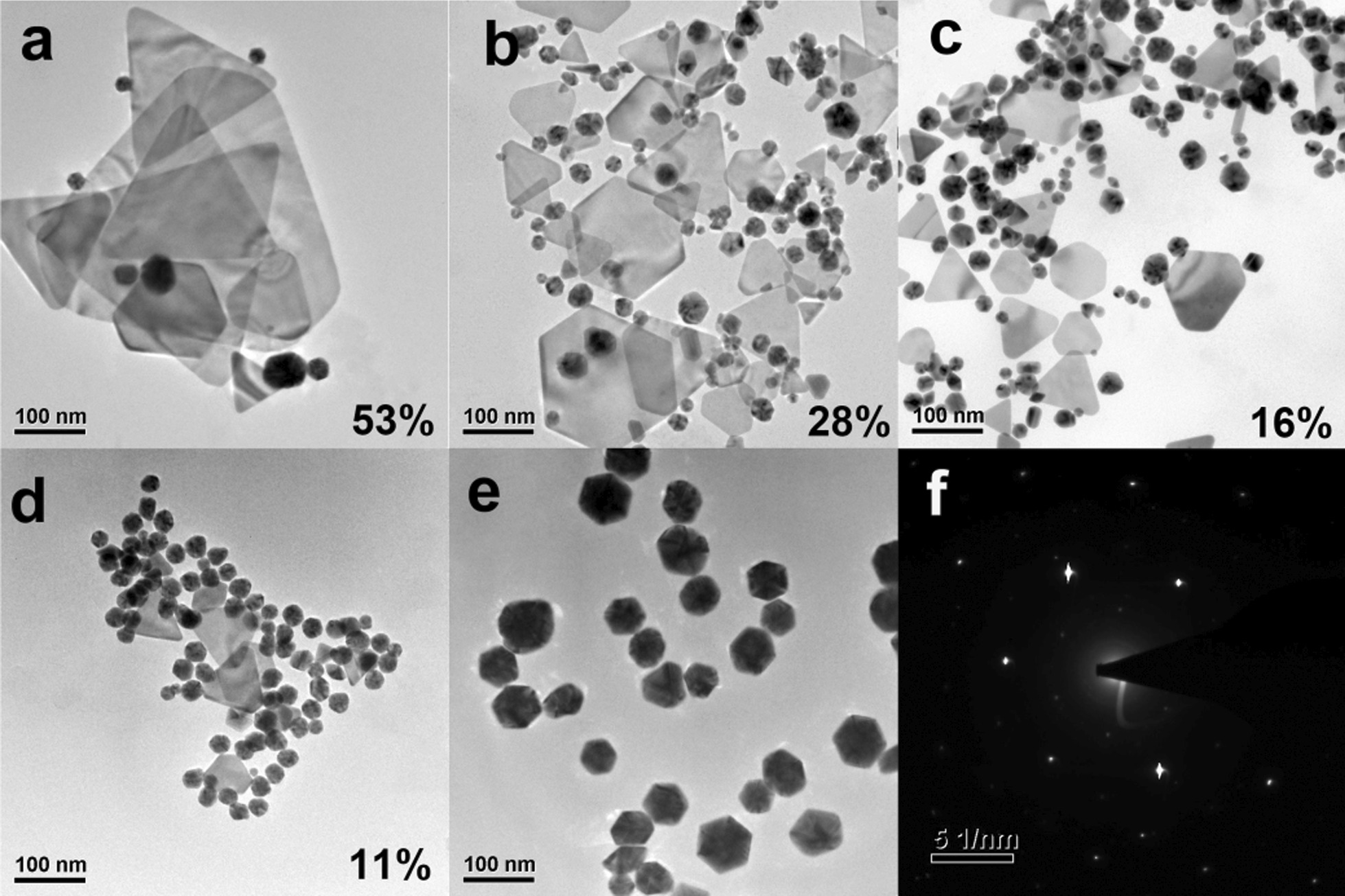
TEM characterizations of GNPs prepared with the feeding rate of 0.5, 1.0, 1.5, 2.0 and 2.5 mL·min^−1^ (**a**–**e**) and SAED pattern of gold nanoplates (**f**)

Figure 2 shows the transmission electron microscope (TEM) images of GNPs synthesized at different conditions. As the feeding rate decreases, the yield of the nanoplates increases from 0 to nearly 53%, and the side length of the nanoplates is about 500 nm. This result shows that a fast release of atoms is not conducive to heterogeneous nucleation, which requires twin seeds and suitable growth rate.

### Formation Mechanism of Gold Nanoplates

**Fig. 3:**
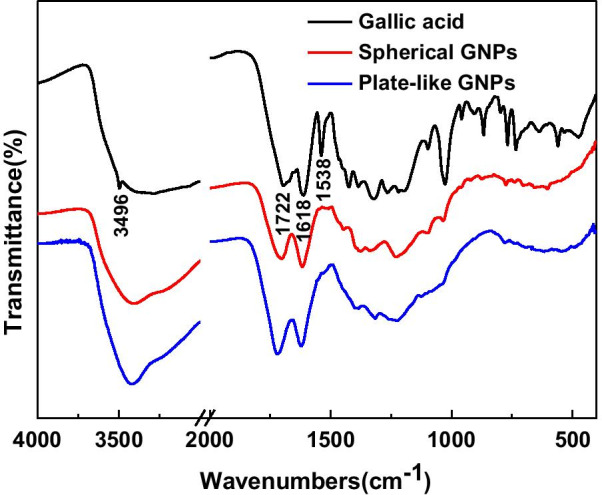
FTIR spectra of gallic acid, spherical and plate-like GNPs

FTIR spectra of gallic acid, spherical and plate-like GNPs are shown in Fig. 3. The peaks at 3496 and 1538 cm^−1^ in the spectrum of gallic acid correspond to the phenolic hydroxyl and benzene ring, which disappear in the spectra of both spherical and plate-like GNPs. This means that gallic acid would not absorb on the nanoparticles. The peaks at 1722 and 1618 cm^−1^ belong to carbonyl group, and carbon–carbon double bonds are observed in both the spherical and plate-like GNPs. The difference is that the absorption of carbonyl group is much stronger in the plate-like nanoparticles. This result indicates that the phenolic hydroxyl was oxidized to the enol structure (absorbed on spherical nanoparticles) and further to the ortho carbonyl compound (absorbed on plate-like nanoparticles).

**Fig. 4:**
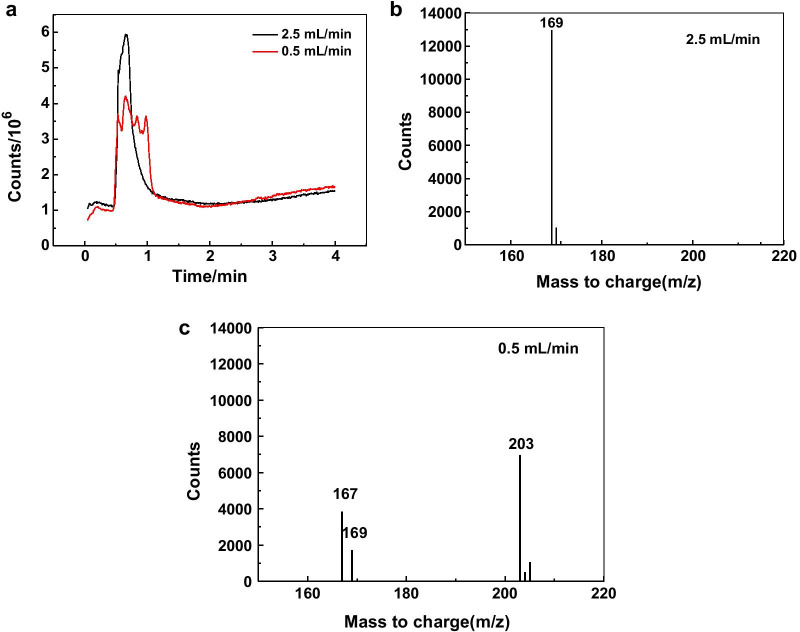
LC–MS analysis of the reaction product of gallic acid

The supernatant of reaction product (5 ml/min and 0. 5 ml/min, respectively) was analyzed by LC–MS. As shown in Fig. 4, for the supernatant obtained from the fast reaction, all the molecules are eluted in about 0.7 min from the column. At this time, the main substances detected by mass spectrometry are molecules with molecular weight of 169; while when the flow rate is slow, the reaction products of gallic acid are relatively complex, and the elution time is 0.5 to 1.1 min. At this time, the m/z of the molecule detected by mass spectrometry is 167, 169, 203, etc. Gallic acid could be oxidized to ortho carbon compounds, and even ortho carbon compounds could be further oxidized to form carboxylic acids. The results of LC–MS analysis showed that the plate-like nanoparticles were formed in a more carbonyl compounds environment.

**Scheme 1:**
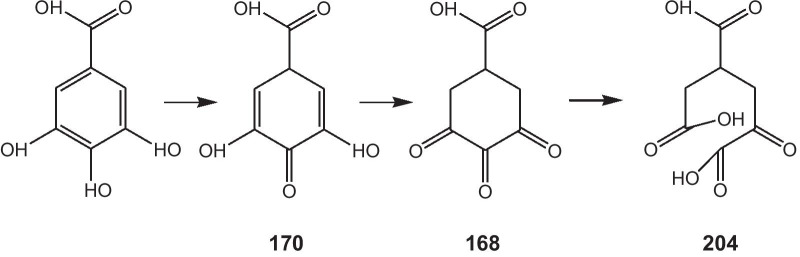
Oxidation of gallic acid to enol and ortho carbonyl compounds

As there were only gallic acid and chloroauric acid in the reaction system, its primary, secondary and further oxidation products (as shown in Scheme 1) might serve as capping reagents and induce the formation of GNPs. At the beginning of the reaction (with a feeding rate of 0.5 mL·min^−1^), chloroauric acid was excessive, gallic acid would be completely oxidized to ortho carbonyl compounds, whereas at a high feeding rate (i.e. 2.5 mL·min^−1^), the gallic acid could be oxidized to enol compounds.

**Fig. 5:**
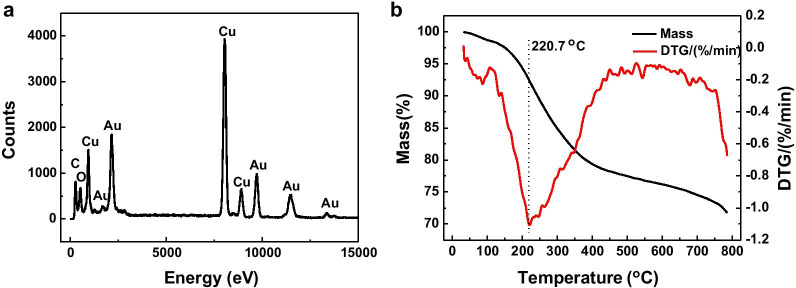
EDS spectrum (**a**) and TG (**b**) profiles of gold nanoplates

In order to clarify the specific adsorption of ortho carbonyl on the gold nanoplates, the adsorbed molecules on the gold nanoplates were studied by EDS (Fig. 5a). Except for Au element, only C and O are found on the surface of gold nanoplates. The ratio of C to O on the surface of the gold nanoplates measured by EDS is 6.8:5 (815:599), close to that in gallic acid (C_7_H_6_O_5_) is 7:5. This indicates that the molecules on the surface of the gold nanoplates are mainly from the oxidation products of gallic acid. TG analysis was conducted to examine the residual molecules on the gold nanoplates. Evidently, Fig. 5b shows that the biomass accounts for 5.6% of the total weight of the gold nanoplates. The decomposition temperature of the biomass is in the range of 400–700 K, in accordance with that of organic matter [34]. This result suggests that the biomass adheres to GNPs as a thin layer and acts as protective agents that prevent the aggregation of the GNPs, which is in agreement with a previous report [35].

**Fig. 6:**
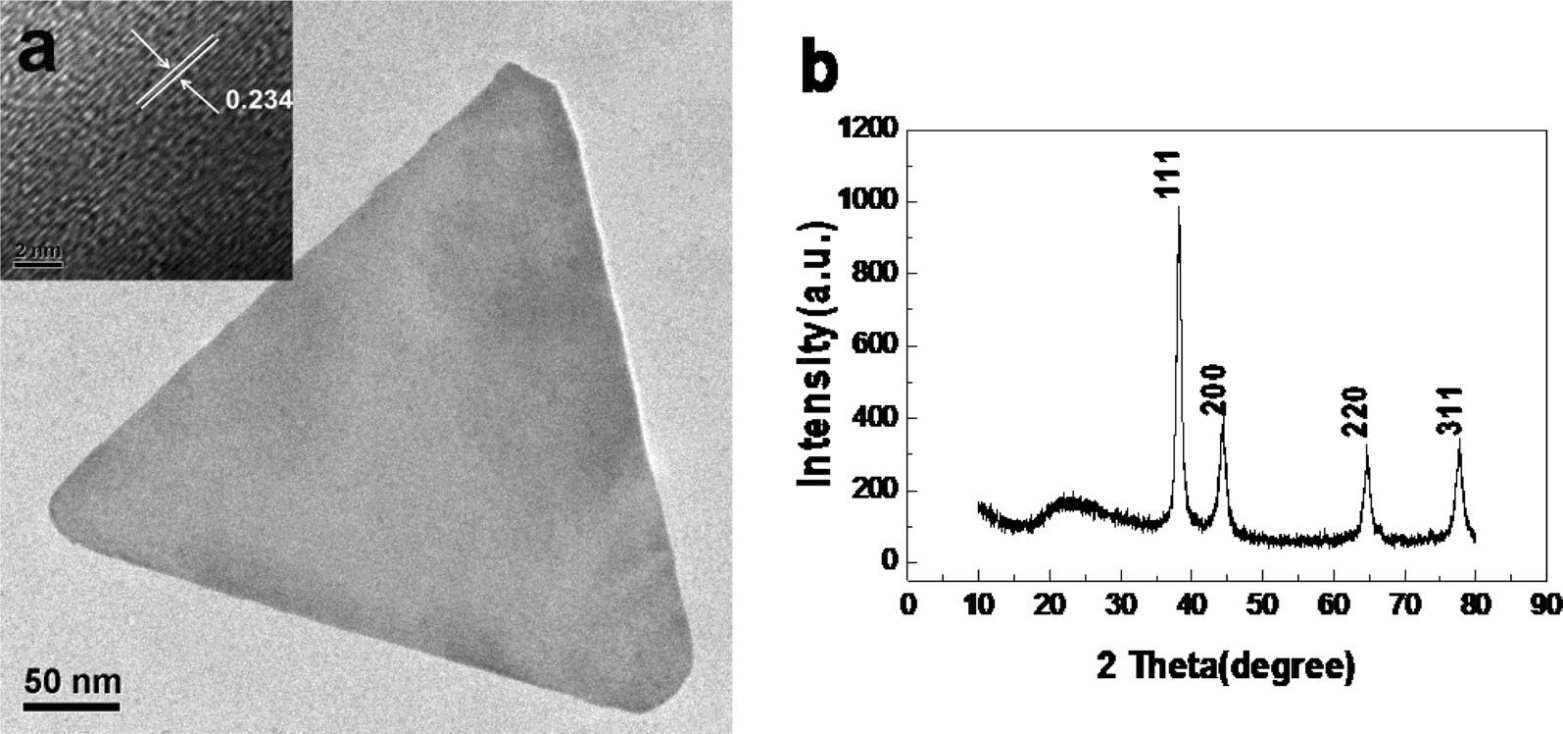
HRTEM (**a**) and XRD (**b**) characterization of gold nanoplates

As we all know, the lattice spacing of different crystal planes is different. For example, the lattice spacing of Au (111) plane is 0.2355 nm and that of (100) plane is 0.408 nm, and the lattice spacing of (110) plane is 0.288 nm. Because of the different arrangement angles between atoms, the bond lengths formed by atoms on different crystal planes are also different. Au (111) plane is the most closely arranged, resulting in the least electronic defects, so the crystal plane energy is the lowest. In this study, the calculated distance between two rows of gold atoms is 0.234 nm (Fig. 6a). The XRD patterns of gold nanoplates (Fig. 6b) show four intense peaks at 38.30°, 44.58°, 64.71° and 77.72°, respectively, which represent the (111), (200), (220) and (311) facets of the face centered cubic crystal structure.

**Fig. 7:**
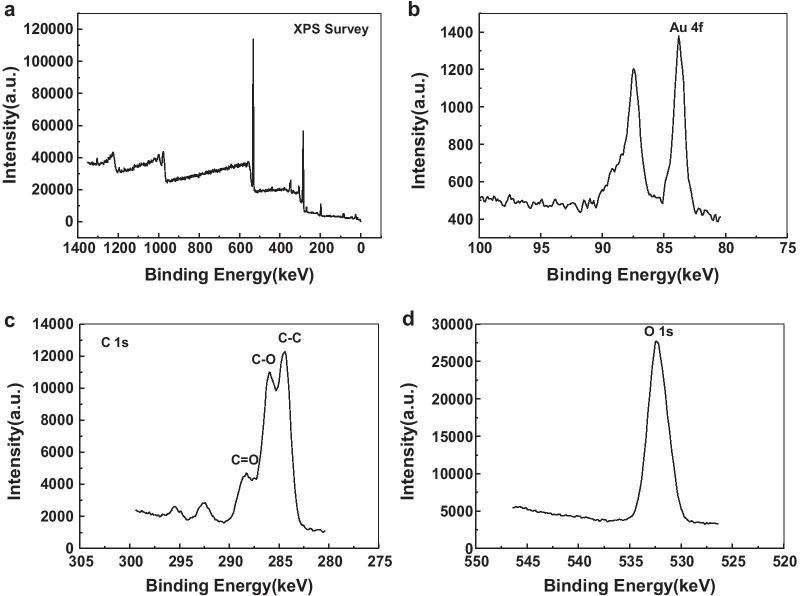
XPS characterization of gold nanoplates

XPS analysis showed that the peaks of Au and O were similar to those reported in most studies [36] (Fig. 7), but the spectra of C were more complex. There were large absorption peaks at 284.5 keV, 286 keV and 288.3 keV, which could be attributed to C–C, C–O and C=O bonds, respectively. XPS spectrum also showed that there were many carbonyl compounds anchored on the surface of the gold nanoplates.

**Scheme 2:**
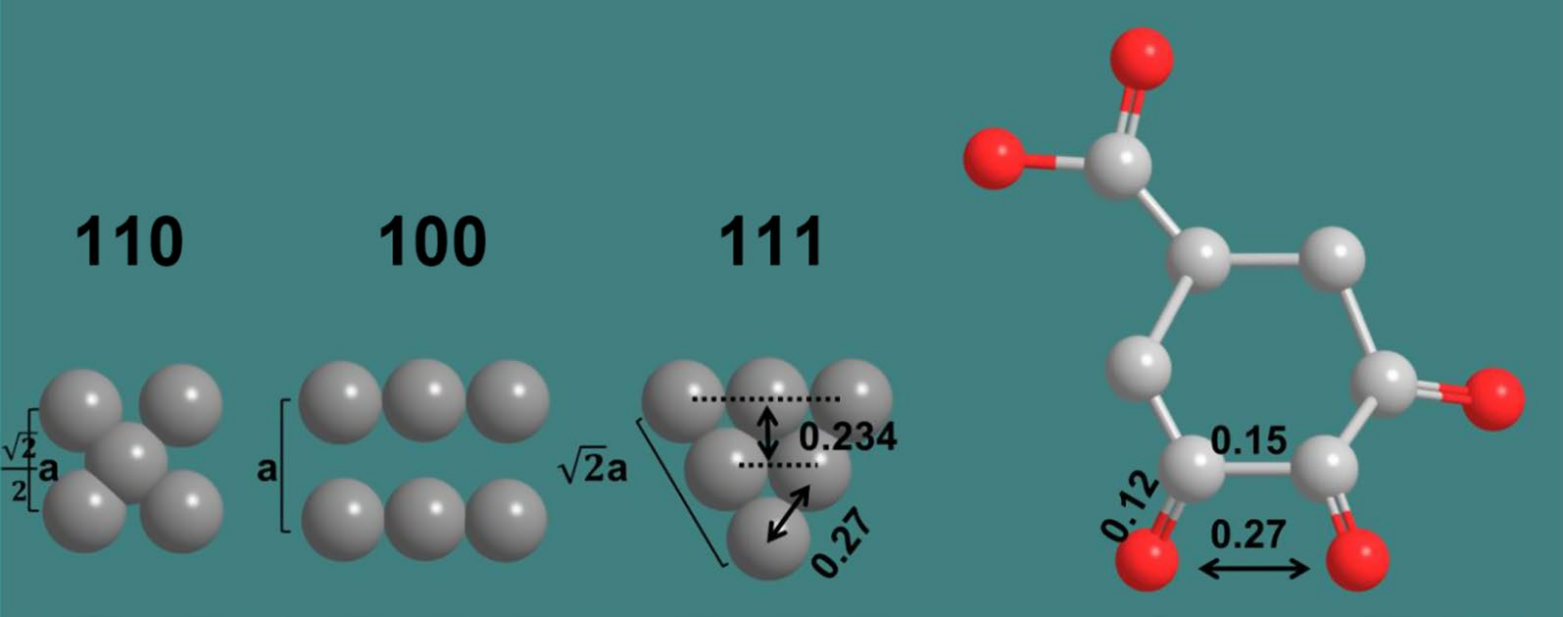
Schematic diagram of the preferential adsorption of capping reagents on Au (111) facet

The primary and secondary oxidation products of gallic acid both have carboxyl and carbonyl group, and the difference is that the latter have ortho carbonyl groups. The bond length of C–C single bond and C=O double bond are 0.15 and 0.12 nm, respectively, whereas the four atoms of ortho carbonyl group form an isosceles trapezoid with a 60° base angle (Scheme 2). Therefore, the distance between the two oxygen atoms can be calculated to be 0.27 nm, which matches the atomic distance of Au (111) planes. This result confirms that the ortho carbonyl group would preferentially adsorb on the surface Au (111) planes to form twin seeds.

### Preparation of Gold Nanoplates with Ortho Carbonyl Compounds as Capping Agents

**Fig. 8:**
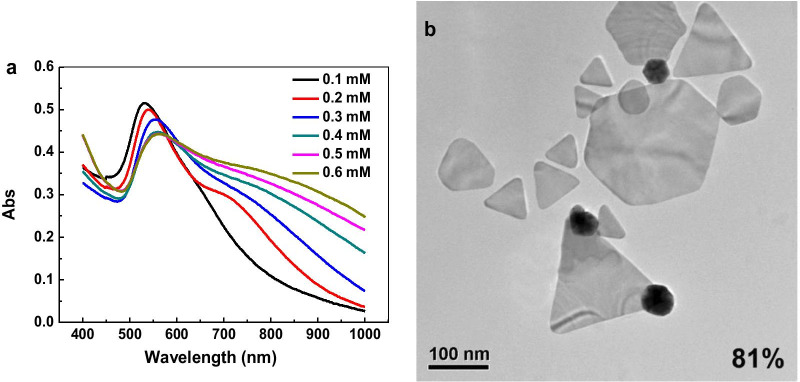
Preparation of GNPs with sodium oxalate as protective agent: **a** UV–Vis spectra; **b** TEM image

To further investigate the effect of ortho carbonyl compounds on the formation of gold nanoplates, sodium oxalate with similar structure was used as a protective agent and ascorbic acid as a reducing agent to prepare GNPs. When the concentration of ascorbic acid was 0.4 mM and the concentration of gold precursor was 1.0 mM. The as-prepared nanoparticles were characterized by UV–Vis spectroscopy (Fig. 8a). As the concentration of sodium oxalate increases from 0.1 to 0.6 mM, the absorption peak of spherical nanoparticles decreases gradually, while the absorption in the long wavelength region increases gradually. Through the TEM characterization, it can be found that when the concentration of sodium oxalate was 0.6 mM, most of the obtained nanoparticles possessed a plate-like morphology (Fig. 8b).

### Electrochemical Sensing of Lead Ions

**Fig. 9:**
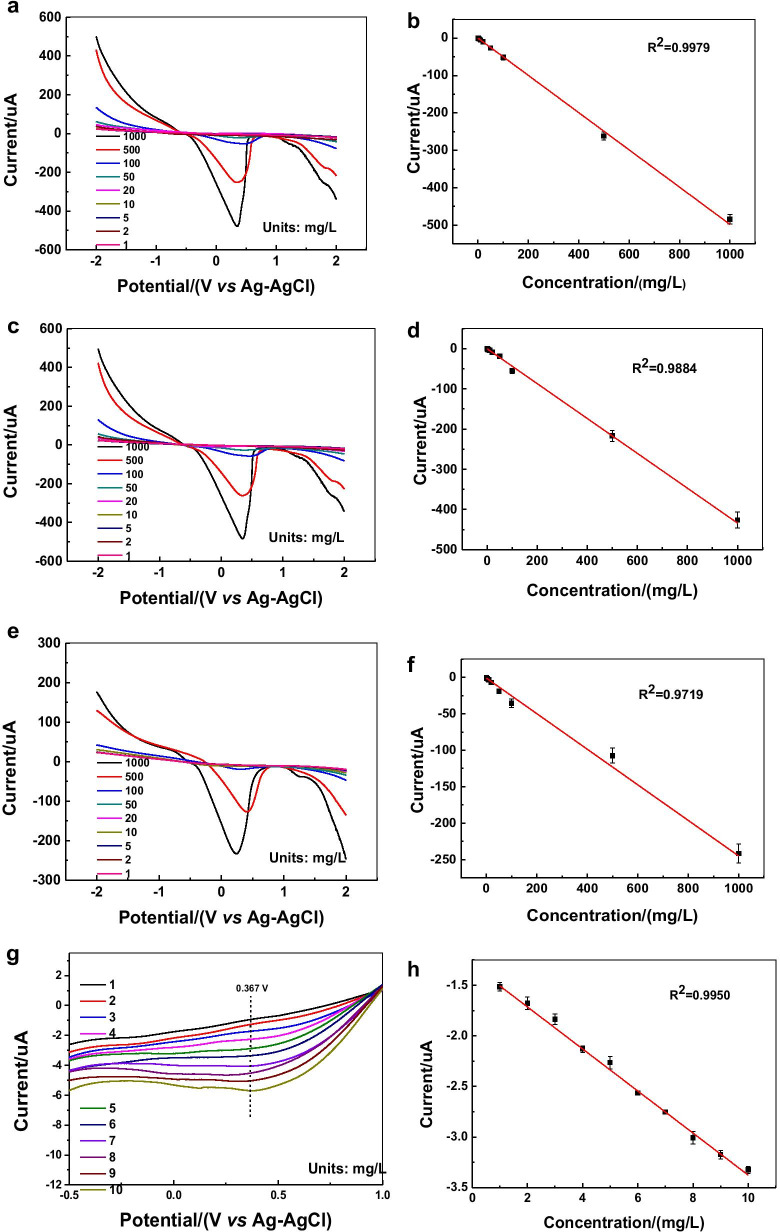
The application of GNPs in the detection of lead ions by linear sweep voltammetry method

**Fig. 10:**
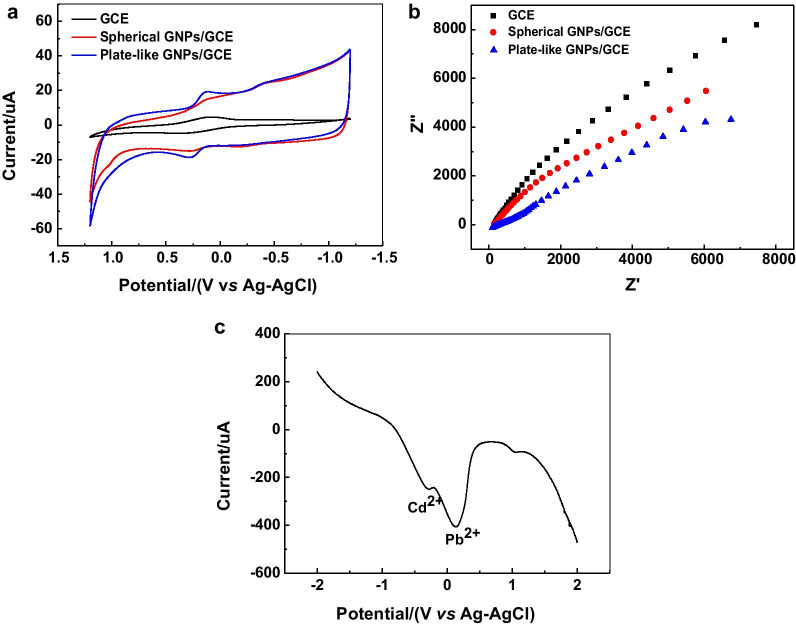
CV (**a**) and EIS (**b**) characterization of the prepared electrodes. **c** Anti-interference performance of the plate-like GNPs/GCE electrode

The electrochemical response of plate-like nanoparticles, spherical nanoparticles and the bare GCE to lead ions is shown in Fig. 9. It can be found that the current response of plate-like nanoparticles to lead ions concentration shows a high linearity (*R*^2^ = 0.9979, Fig. 9a, b), whereas for the spherical GNPs, the linearity between the concentration and the current value is lower (*R*^2^ = 0.9884, Fig. 9c, d). The bare GCE shows an even lower linearity (*R*^2^ = 0.9719, Fig. 9e, f) between the concentration and the current in the concentration range of 1000–10 mg·L^−1^. What is more, the current response of bare GCE is much weaker than that of electrodes loaded with GNPs. The plate-like GNPs have active edges and therefore show amplified signals in the lead ion solution [37, 38]. The durability of gold nanoplates-modified GCE was further evaluated in the electrochemical test of lead ions after being placed at ambient atmosphere for 3 weeks. As shown in Fig. 10g, h, the relationship between the concentration and the current remains a high linearity (*R*^2^ = 0.9950), and this modified electrode is expected to be used in the detection of lead ion concentration in heavy metal wastewater.

To investigate the surface characteristics of the modified electrodes, the prepared electrodes were characterized by CV in 1.0 mM K_3_[Fe(CN)_6_] solution with 0.5 M KCl as a supporting electrolyte. The potential scan ranged from − 1.2 to 1.2 V, and the scan rate was 0.05 V·s^−1^. According to Fig. 10a, a distinct redox peak was detected in the case of bare GCE. When the GCE surface was modified with GNPs, the current response was higher than that of bare GCE. This increase is attributed to the fact that GNPs can promote the electron transfer and improve the conductivity of the electrode. It should be mentioned that the increase of current response would be higher when the GCE was modified by gold nanoplates. What is more, the electrode interface property is also investigated by electrochemical impedance spectroscopy (EIS), and the results are presented in Fig. 10b. The radiuses of the semicircle in Nyquist plot represent the charge transfer resistance (*R*_ct_). The *R*_ct_ of both spherical and plate-like GNPs-modified electrodes is much lower than that of bare GCE, because of the high conductivity of GNPs. Another major problem hindering the accurate detection and identification of lead ions is the interference from other heavy metal ions. The anti-interference performance of the plate-like GNPs/GCE electrode was tested in a mixed solution containing 1.0 g·L^−1^ lead ion and 1.0 g·L^−1^ cadmium ion. As shown in Fig. 10c, there is no obvious change in the potential and current response intensity of the characteristic peak of lead ion, while the difference between the peak positions of cadmium ion and lead ion is 433 mV, indicating that the plate-like GNPs/GCE electrode has good selectivity and anti-interference ability.

**Scheme 3:**
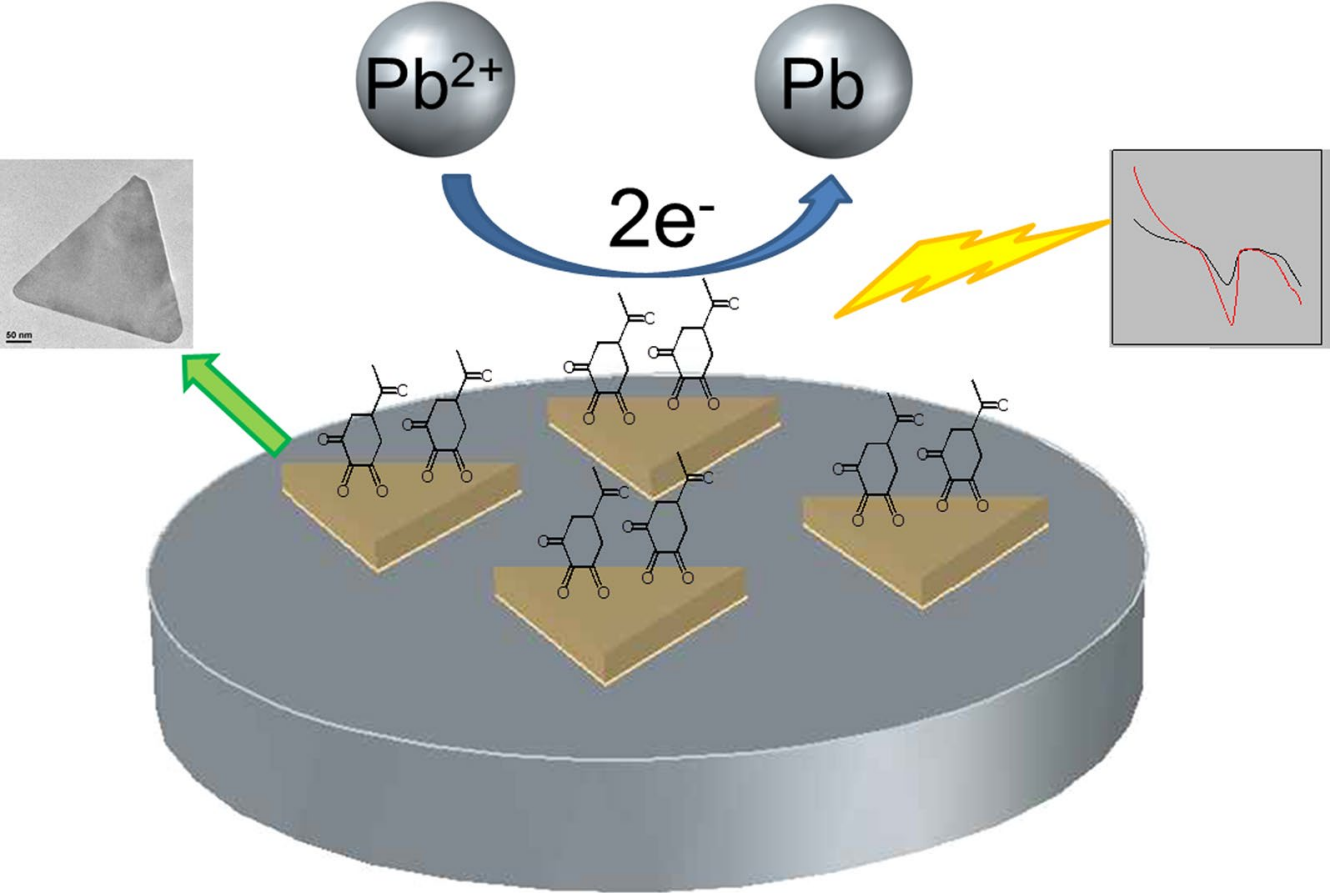
Schematically illustration of the sensing of lead ions by the gold nanoplates-modified GCE

In linear sweep voltammetry, the peak potential is related to the nature of the substance and the supporting electrolyte, while the peak current is linearly related to the concentration of the substance. In this experiment, a bare GCE could detect the concentration of lead ion, but the detection accuracy is low due to the surface passivation and other influence factors. GNPs have good conductivity and special surface properties, which can reduce the impedance of the electrode, thus amplifying the current signal and obtaining better accuracy in the detection of lead ions (Scheme 3). Owing to unique interaction between lead ions and ortho carbonyl compounds, which anchored on the gold nanoplates, it shows a well selectivity with lead ions.

## Conclusions

In conclusion, gold nanoplates have been synthesized by plant molecules. The formation of nanoplates is mainly due to the specific adsorption of ortho carbonyl compounds on the gold (111) facets. The distance between two oxygen atoms matches well with the spacing of gold (111) facet, which is beneficial to the formation of twin seeds and further the growth of plate-like GNPs. Due to the distinctive "edge effect" of gold nanoplates, the signal of lead ions in the linear sweep voltammetry test is much stronger than that of the bare electrode or spherical gold nanoparticle-modified electrode. The developed gold nanoplates are expected to be used in the detection of lead ion concentration in heavy metal wastewater.

## Data Availability

The datasets used and/or analyzed during the current study are available from the co-responding author on reasonable request.

## Abbreviations

LSPR: Localized surface plasmon resonance
GNPs: Gold nanoparticles
TEM: Transmission electron microscopy
HRTEM: High resolution transmission electron microscopy
SAED: Selected area electron diffraction
EDS: Energy-dispersive spectroscopy
FTIR: Fourier transform infrared spectrometer
TG: Thermogravimetry
XRD: X-ray diffractomer
XPS: X-ray photoelectron spectroscopy
LC: Liquid chromatograph
MS: Mass spectrometer
LC–MS: Liquid chromatograph–mass spectrometer
GCE: Glassy carbon electrode
CV: Cyclic voltammetry
EIS: Electrochemical impedance spectroscopy
*R*_ct_: Charge transfer resistance

## References

[1] Stewart ME, Anderton CR, Thompson LB, Maria J, Gray SK, Rogers JA, Nuzzo RG (2008). Nanostructured plasmonic sensors. Chem Rev.

[2] Morarescu R, Shen HH, Vallee RAL, Maes B, Kolaric B, Damman P (2012). Exploiting the localized surface plasmon modes in gold triangular nanoparticles for sensing applications. J Mater Chem.

[3] Major TA, Devadas MS, Lo SS, Hartland GV (2013). Optical and dynamical properties of chemically synthesized gold nanoplates. J Phys Chem C.

[4] Jain PK, Huang X, El-Sayed IH, El-Sayed MA (2008). Noble metals on the nanoscale: optical and photothermal properties and some applications in imaging, sensing, biology, and medicine. Acc Chem Res.

[5] He Q, Liu J, Liu X, Li G, Chen D, Deng P, Liang J (2019). A promising sensing platform toward dopamine using MnO_2_ nanowires/electroreduced graphene oxide composites. Electrochim Acta.

[6] Sun M, Zhai C, Hu J, Zhu M, Pan J (2018). Plasmon enhanced electrocatalytic oxidation of ethanol and organic contaminants on gold/copper iodide composites under visible light irradiation. J Colloid Interface Sci.

[7] Ye P, Xin W, De Rosa I, Wang Y, Goorsky M, Zheng L, Yin X, Xie Y (2020). One-pot self-templated growth of gold nanoframes for enhanced surface-enhanced Raman scattering performance. ACS Appl Mater Interfaces.

[8] Mohammad AM, Al-Akraa IM, El-Deab MS (2018). Superior electrocatalysis of formic acid electro-oxidation on a platinum, gold and manganese oxide nanoparticle-based ternary catalyst. Int J Hydrog Energy.

[9] Saha K, Agasti SS, Kim C, Li X, Rotello VM (2012). Gold nanoparticles in chemical and biological sensing. Chem Rev.

[10] Dreaden EC, Alkilany AM, Huang X, Murphy CJ, El-Sayed MA (2012). The golden age: gold nanoparticles for biomedicine. Chem Soc Rev.

[11] Imaeda K, Hasegawa S, Imura K (2018). Static and dynamic near-field measurements of high-order plasmon modes induced in a gold triangular nanoplate. J Phys Chem Lett.

[12] Jans H, Huo Q (2012). Gold nanoparticle-enabled biological and chemical detection and analysis. Chem Soc Rev.

[13] Imaeda K, Hasegawa S, Imura K (2018). Imaging of plasmonic eigen modes in gold triangular mesoplates by near-field optical microscopy. J Phys Chem C.

[14] Leonardo S, Coronado-Puchau M, Giner-Casares JJ, Langer J, Liz-Marzán LM (2014). Monodisperse gold nanotriangles: size control, large-scale self-assembly, and performance in surface-enhanced Raman scattering. ACS Nano.

[15] Hwang A, Kim E, Moon J, Lee H, Lee M, Jeong J, Lim EK, Jung J, Kang T, Kim B (2019). Atomically flat Au nanoplate platforms enable ultraspecific attomolar detection of protein biomarkers. ACS Appl Mater Interfaces.

[16] Millstone JE, Wei W, Jones MR, Yoo HJ, Mirkin CA (2008). Iodide ions control seed-mediated growth of anisotropic gold nanoparticles. Nano Lett.

[17] Xin W, Severino J, De Rosa IM, Yu D, McKay J, Ye P, Yin X, Yang JM, Carlson L, Kodambaka S (2018). One-step synthesis of tunable-size gold nanoplates on graphene multilayers. Nano Lett.

[18] Yang F, Huang J, Odoom-Wubah T, Hong Y, Du M, Sun D, Jia L, Li Q (2015). Efficient Ag/CeO_2_ catalysts for CO oxidation prepared with microwave-assisted biosynthesis. Chem Eng J.

[19] Shaik F, Zhang W, Niu W (2017). A novel photochemical method for the synthesis of Au triangular nanoplates inside nanocavity of mesoporous silica shells. J Phys Chem C.

[20] Nori NM, Abdi K, Khoshayand MR, Ahmadi SH, Lamei N, Shahverdi AR (2013). Microwave-assisted biosynthesis of gold-silver alloy nanoparticles and determination of their Au/Ag ratio by atomic absorption spectroscopy. J Exp Nanosci.

[21] Personick ML, Mirkin CA (2013). Making sense of the mayhem behind shape control in the synthesis of gold nanoparticles. J Am Chem Soc.

[22] Hormozi-Nezhad MR, Karami P, Robatjazi H (2013). A simple shape-controlled synthesis of gold nanoparticles using nonionic surfactants. RSC Adv.

[23] Moon SY, Kusunose T, Sekino T (2009). CTAB-assisted synthesis of size- and shape-controlled gold nanoparticles in SDS aqueous solution. Mater Lett.

[24] Iravani S (2011). Green synthesis of metal nanoparticles using plants. Green Chem.

[25] Mazdeh SK, Motamedi H, Khiavi AA, Mehrabi MR (2014). Gold nanoparticle biosynthesis by *E. coli* and conjugation with streptomycin and evaluation of its antibacterial effect. Curr Nanosci.

[26] Huang J, Li Q, Sun D, Lu Y, Su Y, Yang X, Wang H, Wang Y, Shao W, He N, Hong J, Chen C (2007). Biosynthesis of silver and gold nanoparticles by novel sundried *Cinnamomum camphora* leaf. Nanotechnology.

[27] Shankar SS, Rai A, Ankamwar B, Singh A, Ahmad A, Sastry M (2004). Biological synthesis of triangular gold nanoprisms. Nat Mater.

[28] Montes MO, Mayoral A, Deepak FL, Parsons JG, Jose-Yacaman M, Peralta-Videa JR, Gardea-Torresdey JL (2011). Anisotropic gold nanoparticles and gold plates biosynthesis using alfalfa extracts. J Nanopart Res.

[29] Zhan G, Ke L, Li Q, Huang J, Hua D, Ibrahim AR, Sun D (2012). Synthesis of gold nanoplates with bioreducing agent using syringe pumps: a kinetic control. Ind Eng Chem Res.

[30] Zhou Y, Lin W, Yang F, Fang W, Huang J, Li Q (2014). Insights into formation kinetics of gold nanoparticles using the classical JMAK model. Chem Phys.

[31] Jiang X, Sun D, Zhang G, He N, Liu H, Huang J, Odoom-Wubah T, Li Q (2013). Investigation of active biomolecules involved in the nucleation and growth of gold nanoparticles by *Artocarpus heterophyllus Lam* leaf extract. J Nanopart Res.

[32] Li S, Li S, Wen N, Wei D, Zhang Y (2021). Highly effective removal of lead and cadmium ions from wastewater by bifunctional magnetic mesoporous silica. Sep Purif Technol.

[33] Chen H, Li W, Wang J, Xu H, Liu Y, Zhang Z, Li Y, Zhang Y (2019). Adsorption ofcadmium and lead ions by phosphoric acid-modified biochar generated from chicken feather: selective adsorptionand influence of dissolved organic matter. Bioresour Technol.

[34] Huang J, Wang W, Lin L, Li Q, Lin W, Li M, Mann S (2009). A general strategy for the biosynthesis of gold nanoparticles by traditional Chinese medicines and their potential application as catalysts. Chem Asian J.

[35] Ah CS, Yun YJ, Park HJ, Kim WJ, Ha DH, Yun WS (2005). Size-controlled synthesis of machinable single crystalline gold nanoplates. Chem Mater.

[36] Liu YB, Yao QF, Wu XJ, Chen TK, Ma Y, Ong CN, Xie JP (2016). Gold nanocluster sensitized TiO_2_ nanotube arrays for visible-light driven photoelectrocatalytic removal of antibiotic tetracycline. Nanoscale.

[37] Xu X, Luo J, Liu M, Wang Y, Yi Z, Li X, Yi Y, Tang Y (2015). The influence of edge and corner evolution on plasmon properties and resonant edge effect in gold nanoplatelets. Phys Chem Chem Phys.

[38] Xu X, Yi Z, Li X, Wang Y, Liu J, Luo J, Luo B, Yi Y, Tang Y (2013). Tunable nanoscale confinement of energy and resonant edge effect in triangular gold nanoprisms. J Phys Chem C.

